# The Burgeoning HIV/HCV Syndemic in the Urban Northeast: HCV, HIV, and HIV/HCV Coinfection in an Urban Setting

**DOI:** 10.1371/journal.pone.0064321

**Published:** 2013-05-14

**Authors:** Jamie P. Morano, Britton A. Gibson, Frederick L. Altice

**Affiliations:** 1 Department of Internal Medicine, Section of Infectious Diseases, AIDS Program, Yale School of Medicine, New Haven, Connecticut, United States of America; 2 Division of Epidemiology of Microbial Diseases, Yale School of Public Health, New Haven, Connecticut, United States of America; Public Health Agency of Barcelona, Spain

## Abstract

**Introduction:**

Despite recommendations for generation-based HCV and once lifetime HIV screening, thousands of individuals in the U.S. still remain untested and undiagnosed. This cross-sectional study examines the correlates of HCV and HIV monoinfection and HIV/HCV coinfection in an urban Northeast setting.

**Methods:**

Utilizing an electronic database from a mobile medical clinic in New Haven, CT from January 2003 to July 2011, 8,311 individuals underwent structured health assessment and screening for HIV and HCV.

**Results:**

HIV [N = 601 (8.0%)] and HCV [N = 753 (10.1%)] infection were identified, and 197 (26.1%) of the 753 with HCV were coinfected with HIV. Both monoinfection and coinfection status were independently correlated with crack cocaine use and increasing age. HIV/HCV coinfection was correlated with men having sex with men (MSM) (AOR = 38.53, p<0.0080), shooting gallery use (AOR = 3.06, p<0.0070), and not completing high school (AOR = 2.51, p<0.0370). HCV monoinfection correlated with health insurance (AOR = 2.16, p<0.0020), domestic violence (AOR = 1.99, p<0.0070), and being Hispanic (AOR = 2.63, p<0.0001), while HIV monoinfection correlated with having had syphilis (AOR = 2.66, p<0.0001) and being Black (AOR = 1.73, p = 0.0010).

**Conclusions:**

Though HIV and HCV share common transmission risk behaviors, independent correlates with viral infection status in an urban Northeast setting are distinct and have important implications for surveillance, healthcare delivery, disease prevention, and clinical care.

## Introduction

Hepatitis C (HCV) and HIV represent two highly prevalent chronic viral infections worldwide and the two most prevalent chronic viral infections in the United States [1], [2]. In the U.S., the Centers for Disease Control and Prevention (CDC) estimates upwards of 4.9 million persons infected with HCV [1], but household surveys do not include the high risk homeless or criminal justice populations. Among the 1.2 million people living with HIV/AIDS (PLWHA), HCV prevalence varies by risk factor and has been documented as high as 80.8% among HIV-infected people who inject drugs (PWIDs), 29.9% among women, and 10.2% among men-who-have-sex-with-men (MSM) [3]. Coinfection complicates clinical care and morbidity and mortality. Internationally, HIV/HCV coinfection is associated with a 6.7-fold increased relative risk of liver-related death compared to those with HIV monoinfection [4]. Since 2007, HCV-related mortality has eclipsed HIV-related mortality [5] and is currently the leading cause of liver transplantation in the United States [6]. HIV infection accelerates hepatic fibrosis in HCV-infected patients, and HCV portends a three-fold likelihood of liver-associated toxicity from antiretroviral therapy (ART) [6]. As a result, HCV screening among PLWHA is recommended, primarily to address medical comorbidity and because both chronic infections are treatable, especially with emerging new treatment options [7].

In a recent CDC study of 7,618 HIV-infected patients in eight urban U.S. clinical care settings, nearly one-quarter (24.2%) of tested patients were HIV/HCV coinfected and correlated with being female, Black and Hispanic race/ethnicity, PWIDs, and those ≥45 years old [3]; however, no sites from the Northeast were included. Few urban samples, moreover, have included high levels of socially and medically marginalized populations, including the homeless [8], released prisoners [9], soup kitchens [10], sexually transmitted infection (STI) clinics [11], or those currently incarcerated [12]. Specifically, real-world estimates are lacking for at-risk, heterogeneous urban Northeastern US populations, including among PWIDs that remain estranged from traditional healthcare settings [13]. This study was undertaken to characterize HIV/HCV coinfection among marginalized groups that do not typically present to traditional care settings.

## Methods

### Study Setting

New Haven, Connecticut, the fourth poorest city for its size, is profoundly impacted by poverty, incarceration, substance use disorders (SUDs), and HIV/AIDS and provides an important setting to assess community-based correlates of HIV/HCV coinfection in an urban setting [14]. The Community Health Care Van (CHCV), a free mobile healthcare program operating since 1993 provides a number of clinical and outreach health care services including screening for HIV and STI, [15]–[20] screening and vaccination for preventable infections [17], treatment of SUDs using buprenorphine and extended-release naltrexone (XR-NTX) treatment for opioid dependence [9], [21]–[23], and XR-NTX for alcoholism, tuberculosis screening and isoniazid preventive treatment [24], syringe distribution, directly administered antiretroviral therapy [16], [18], [25], [26], and a variety of case management [27] and post-prison release services [28]. Dedicated bilingual medical and counseling staff members provide weekday services in four distinct impoverished neighborhoods within New Haven, CT.

Despite the large proportion of CHCV clients with health insurance, many clients seek clinical care on the CHCV as its services are culturally competent, targeted for community outreach, geographically convenient, and free of charge. Similarly, CHCV services are confidential, delivered without stigma at the doorsteps of patients, and include non-judgmental counseling and testing for a variety of sensitive medical and mental health conditions, including crisis interventions for assault and substance use disorders.

Compared to the general New Haven population of 130,000, CHCV clients are more likely to be more foreign born (36.3% vs 16.7%), with fewer completing high school (27.7% vs 18.5%), and more likely to be of minority racial/ethnic background (40.0% vs 33.4% for African Americans and 34.4% vs 27.4% for Hispanics) [29]. CHCV clients had an 8.8% HIV prevalence and 9.9% prevalence of Hepatitis C, compared to a city wide prevalence of 1.1% for HIV [30] and 0.7% for Hepatitis C [31], although HCV reporting is less robust.

### Patient Inclusion

The CHCV implemented an electronic clinical database starting in 2003 that includes all standardized health assessments. All clients are routinely screened for risk factors and tested for HIV and HCV infections. Risk questions relate to drug injection, syringe exchange practices, oral and inhaled drug use, sexual risk behaviors, sexual orientation, engagement in transactional sex and sex solicitation, and demographic and social characteristics. Experience with domestic violence, sexual assault, insurance status, and healthcare utilization is also recorded. All clinical data were recorded using standardized instruments and electronically scanned using Teleform (Cardiff Digital Documents, Cherry Valley, CA) and were verified for content before incorporation into a secure electronic clinical database. Clients were routinely tested for HIV and risk-based testing was conducted for HCV.

This analysis of existing clinical data was granted exemption status because all data were de-identified, observational, and deemed part of non-experimental, routine medical care. No identities were available within the dataset, and de-identified data were stored on password-protected servers. The study was conducted under the purview of the ethics committee at Yale University School of Medicine and was reviewed and granted an exemption by the Yale Human Investigations Committee IRB.

### Definitions

“Baby boomer” generation, a new CDC risk factor for HCV infection, was defined as being born from 1945 through 1965. Stable housing was defined as living in one's own apartment, one's own house, or with one's family, while unstable housing was defined as living with a friend, in a hotel or shelter, or on a street or public place. Being in a relationship was defined as being married or cohabitating with a significant other. Employment and health insurance status was self-reported. Mental health comorbidity was counted if the client recorded a history of anxiety, depression, bipolar disorder, post-traumatic stress disorder (PTSD), schizophrenia, or other known prior mental health issue. Standardized measures of type, route, and frequency of drug use were assessed using the drug component of the Addiction Severity Index [32]. Hazardous drinking was defined as three or more drinks daily for men and two or more drinks daily for women; a “drink” was defined as the equivalent to 12 ounces of beer or 1.5 ounces of hard liquor. An injected mixture of heroin and cocaine is known as “speedball.” The oral drug MDMA (3,4-methylenedioxy-N-methylamphetamine) is known as “ecstasy.” Opioid replacement history was defined as ever having participated in a methadone or buprenorphine program. Opioid dependence was defined as a client reporting history of dependence on any opioid according to DSM-IV criteria. Transactional sex is defined as exchanging sexual intercourse for money, drugs, rent, or protection. Sex solicitation is defined as paying money specifically for sexual intercourse.

### Eligibility

From January 2003 until July 2011, 8,312 unique individuals were identified among 23,628 CHCV visits. Overall 7,473 (89.9%) clients were included in the final analysis because they had completed the full initial intake assessment during the observation period. HIV/HCV coinfection, HIV monoinfection, and HCV monoinfection were defined objectively by documentation by antibody status for HIV or HCV. Additionally, having a documented viral load, CD4 count, or prescribed ART regimen confirmed HIV status. A self-reported previous positive test as a reason for not undergoing antibody testing was also included.

Newly identified HIV-infected patients were counseled and underwent immediate phlebotomy for confirmatory Western Blot testing, viral load and CD4 count assessments, and direct linkage to a HIV provider. Newly identified HCV-infected individuals were assessed by standard phlebotomy screens and referred for further follow-up laboratory panels with referral to the appropriate HCV provider. Complete laboratory findings were not included in the analysis due to the multiple laboratory testing sites in the community.

### Analytic Strategy

All data were verified using SPSS (PASW Statistics Release 18.0, Armonk, NY, USA) and then analyzed using STATA IC 12 (College Station, TX, USA). All clients have a unique clinical code, but unique identifiers were removed before analyzing data. Compared to those without any documented infection (N = 6375), three groups were defined for this analysis: 1) HIV/HCV coinfection (N = 193); 2) HCV monoinfection (N = 546); and 3) HIV monoinfection (N = 359). The Kruskal-Wallis test p-value was used to assess difference between the four diagnostic groups assuming a non-parametric population distribution. The findings for the main outcomes were confirmed and remained robust when using the Dunn, Bonferroni, and Scheffe multiple corrections tests, except where indicated in the text [33]. The Levene test, robust for non-parametric distributions, was also found to be consistent [34]. Percentages are out of column totals and may not add to 100% due to non-response and rounding.

Bivariate logistic regression was first performed to identify the correlations of each of the three dependent variables: (1) HIV/HCV coinfection, (2) HCV monoinfection, and (3) HIV monoinfection. Due to the need to control for confounding variables, a multivariate logistic regression for all correlates that were significant at the p<0.05 level were included in the final models. Ultimately, both forward and backward step-wise regression models using a criterion of p<0.05 were utilized to establish the best fit model of HIV/HCV coinfection with comparison best fit models of both HIV and HCV monoinfection. Goodness-of-fit testing was assessed using the Akaike Information Criteria (AIC), Bayesian Information Criterion (BIC), and the Pseudo R^2^ methods.

To inform the geographical distribution of HCV and HIV infection, addresses were mapped for spatial analysis using ArcGIS 9.1 (Redlands, CA, USA). Clients who provided any address in Connecticut, including a shelter or hotel address, were included in this analysis. Addresses were aggregated to census block group to preserve client anonymity.

In order to assess the geospatial density for each dependent variable, prevalence for HIV/HCV coinfection, HCV monoinfection, and HIV monoinfection was calculated as the number of infected clients per total number of individual CHCV clients residing in each census block group. These data were mapped, and census block group prevalence was represented using a five-tiered gradient.

## Results

Of the total 8,311 unique patient data included, 753 (10.1%) and 601 (8.0%) were HCV- and HIV-infected, respectively. Among the 753 with HCV infection, 197 (26.1%) were coinfected with HIV. Similarly, 32.1% of HIV-infected patients were coinfected with HCV. Though these estimates are the minimum prevalence of infection because not all patients accepted testing using our routine opt-out strategy, HIV and HCV monoinfection in this urban setting was estimated at 4.8% and 7.3%, respectively, while HIV/HCV coinfection prevalence was estimated at 2.6%.

Analysis of the 7,473 clients with complete information (89.9% of total sample) is shown in Table 1 with HIV/HCV coinfected individuals (N = 193), HCV monoinfected individuals (N = 546), and HIV monoinfected individuals (N = 359). Geographic distribution of prevalence by New Haven census block were successfully mapped for clients with completed survey data with available addresses for HIV/HCV coinfection, HCV monoinfection, and HIV monoinfection (N = 7,720). Patients with HIV/HCV coinfection differed significantly to the HIV and HCV monoinfected groups for a number of different behavioral, social, and demographic characteristics (Table 1). Specifically, HIV/HCV coinfected individuals were more likely to share membership with the “baby boomer” generation (96.7%), be US-born (82.9%), be unemployed (92.2%), had a previous STI (37.8%), and had been diagnosed with mental illness (54.4%) (all p<0.0001). In addition, the HIV/HCV coinfected group was significantly more likely to be comprised of PWIDs (87.6%), including heroin (88.1%), cocaine (89.6%), speedball injection (69.4%), as well as crack cocaine use (82.4%), and were more likely to be opioid dependent (42.0%) and ever receiving opioid replacement therapy, including methadone or buprenorphine therapy (45.6%) (p<0.0001 for all comparisons). Transactional sex, including women soliciting sex (2.6%) and men being paid for sex (14.5%), was also associated with increased HIV/HCV coinfection (all p<0.0001).

**Table 1:** Characteristics among patients with completed surveys with HIV/HCV coinfection as compared to patients with HIV monoinfection, HCV monoinfection, and those neither infected with HIV nor HCV.

	Total N = 7473	HIV/HCV coinfected N = 193	HCV mono-infected N = 546	HIV mono-infected N = 359	Non-Infected N = 6375	p-value^a^
**Number of Completed Surveys**	**7473**	**193**	**546**	**359**	**6375**	
Mean Age, years	36.9	48.4*	41.2*	41.3*	35.5	<0.0001
“Baby Boomer” Generation	2526 (33.8%)	187 (96.7%)*	264 (48.4%)*	149 (41.5%)*	1926 (30.2%)	<0.0001
Gender						
Male	4165 (55.7%)	136 (70.5%)*	339 (62.1%)	202 (56.3%)	3488 (54.7%)	<0.0001
Female	3308 (44.3%)	57 (29.5%)	207 (37.9%)	157 (43.7%)	2887 (45.3%)	
Race/Ethnicity						
White	1836 (24.6%)	54 (28.0%)	280 (51.3%)*	63 (17.5%)	1439 (22.6%)	<0.0001
Black	2992 (40.0%)	80 (41.5%)	76 (13.9%)*	183 (51.0%)	2653 (41.6%)	<0.0001
Hispanic Ethnicity ^b^	2579 (34.5%)	59 (30.6%)	188 (34.4%)	109 (30.4%)	2223 (34.9%)	0.2191
Other ^b^	66 (0.9%)	0 (0.0%)	2 (0.4%)	4 (1.1%)	60 (0.9%)	0.2762
U.S. Born						
Yes	5400 (72.3%)	160 (82.9%)	494 (90.4%)*	244 (68.0%)*	4502 (70.6%)	<0.0001
No	2073 (27.7%)	33 (17.1%)	52 (9.5%)	115 (32.0%)	1873 (29.4%)	
High School Level Completed ^b^						
Yes	5161 (69.1%)	112 (58.0%)	366 (67.0%)	237 (66.0%)	4446 (69.7%)	0.1283
No	2099 (28.1%)	75 (38.9%)	169 (31.0%)	117 (32.6%)	1738 (27.3%)	
Stable Housing						
Yes	5018 (67.1%)	82 (42.5%)*	214 (39.2%)*	229 (63.8%)	4493 (70.5%)	<0.0001
No	2396 (32.1%)	107 (55.4%)	332 (61.1%)	123 (34.3%)	1834 (28.8%)	
In a Relationship						
Yes	1479 (19.8%)	24 (12.4%)	64 (11.7%)*	87 (24.2%)	1304 (20.5%)	<0.0001
No	5968 (79.9%)	164 (85.0%)	480 (87.9%)	267 (74.4%)	5057 (79.3%)	
Employed						
Yes	2391 (32.0%)	10 (5.2%)*	57 (10.4%)*	80 (22.3%)*	2244 (35.2%)	<0.0001
No	5028 (67.3%)	178 (92.2%)	489 (89.6%)	274 (76.3%)	4087 (64.1%)	
Health Insurance						
Yes	3327 (44.5%)	130 (67.4%)*	405 (74.2%)*	187 (52.1%)*	2605 (40.8%)	<0.0001
No	4071 (54.5%)	58 (30.0%)	139 (25.5%)	168 (46.8%)	3706 (58.1%)	
Injection Drug Use (PWID)						
Yes	1235 (16.5%)	169 (87.6%)*	441 (80.8%)*	70 (19.5%)	555 (8.7%)	<0.0001
No	6238 (83.5%)	24 (12.4%)	105 (19.2%)	289 (80.5%)	5820 (91.3%)	
Injection Drug Use, Last 30 Days						
Yes	388 (5.2%)	45 (23.3%)*	156 (28.6%)*	18 (5.0%)	169 (2.7%)	<0.0001
No	5988 (80.1%)	142 (73.4%)	375 (68.7%)	255 (71.0%)	5216 (81.8%)	
Non-injection Drug Use, excluding Marijuana ^b^						
Yes	239 (3.2%)	5 (2.6%)	9 (1.6%)	18 (5.0%)	207 (3.2%)	0.0396
No	7234 (96.8%)	188 (97.4%)	537 (98.3%)	341 (95.0%)	6168 (96.7%)	
Needle Exchange Program Use						
Yes	369 (4.9%)	57 (29.5%)*	168 (30.8%)*	19 (5.3%)	125 (2.0%)	<0.0001
No	3718 (49.7%)	73 (37.8%)	243 (44.5%)	161 (44.8%)	3241 (50.8%)	
Needle Exchange Program, Last 30 Days ^b^						
Yes	103 (1.38%)	14 (7.2%)	45 (8.2%)	6 (1.7%)	38 (0.6%)	0.7329
No	526 (7.04%)	60 (31.1%)	206 (37.7%)	20 (5.6%)	240 (3.8%)	
Heroin Use						
Yes	1771 (23.7%)	170 (88.1%)*	471 (86.3%)*	116 (32.3%)*	1014 (15.9%)	<0.0001
No	5702 (76.3%)	23 (11.9%)	75 (13.7%)	243 (67.7%)	5361 (84.1%)	
Injected with Used Needles						
Yes	336 (4.5%)	53 (27.5%)*	170 (31.1%)*	11 (3.1%)	102 (1.6%)	<0.0001
No	457 (6.1%)	36 (18.7%)	130 (23.8%)	18 (5.0%)	273 (4.3%)	
Shooting Gallery Use						
Yes	211 (2.8%)	43 (22.3%)*	116 (21.2%)*	5 (1.4%)	47 (0.7%)	<0.0001
No	338 (4.5%)	28 (14.5%)	121 (22.2%)	10 (2.8%)	179 (2.8%)	
Cocaine Use						
Yes	2575 (34.5%)	173 (89.6%)*	457 (83.7%)*	164 (45.7%)*	1781 (27.9%)	<0.0001
No	4898 (65.5%)	20 (10.4%)	89 (16.3%)	195 (54.3%)	4594 (72.1%)	
Crack Cocaine Use						
Yes	2190 (29.3%)	159 (82.4%)*	426 (78.0%)*	165 (46.0%)*	1440 (22.6%)	<0.0001
No	5283 (70.7%)	34 (17.6%)	120 (22.0%)	194 (54.0%)	4935 (77.4%)	
Marijuana Use						
Yes	4630 (62.0%)	172 (89.1%)*	481 (88.1%)*	244 (68.0%)	3733 (58.5%)	<0.0001
No	2843 (38.1%)	21 (10.9%)	65 (11.9%)	115 (32.0%)	2642 (41.4%)	
Combined Cocaine and Heroin Injection (“Speedball”) Use						
Yes	911 (12.2%)	134 (69.4%)*	289 (52.9%)*	66 (18.4%)*	422 (6.6%)	<0.0001
No	6562 (87.8%)	59 (30.6%)	257 (47.1%)	293 (81.6%)	5953 (93.4%)	
Crystal Methamphetamine Use						
Yes	274 (3.7%)	29 (15.0%)*	89 (16.3%)*	12 (3.3%)	144 (2.3%)	<0.0001
No	7199 (96.3%)	164 (85.0%)	457 (83.7%)	347 (96.7%)	6231 (97.7%)	
MDMA (“Ecstasy”) Use						
Yes	794 (10.6%)	19 (9.8%)	115 (21.1%)*	31 (8.6%)	629 (9.9%)	<0.0001
No	6679 (89.4%)	174 (90.2%)	431 (78.9%)	328 (91.4%)	5746 (90.1%)	
Hazardous Drinking ^b^						
Yes	1370 (18.3%)	37 (19.2%)	83 (15.2%)	74 (20.6%)	1176 (18.4%)	0.1742
No	6103 (81.7%)	156 (80.8%)	463 (84.8%)	285 (79.4%)	5199 (81.6%)	
Opioid Dependence						
Yes	658 (8.8%)	81 (42.0%)*	163 (29.9%)*	52 (14.5%)*	362 (5.7%)	<0.0001
No	6815 (91.4%)	112 (58.0%)	383 (70.1%)	307 (85.5%)	6013 (94.3%)	
Opioid Replacement Use						
Yes	641 (8.6%)	88 (45.6%)*	194 (35.5%)*	50 (13.9%)*	309 (4.8%)	<0.0001
No	6832 (91.4%)	105 (54.4%)	352 (64.5%)	309 (86.1%)	6066 (95.2%)	
Emergency Room Past 6 Months						
Yes	2283 (30.5%)	86 (44.6%)*	251 (46.0%)*	118 (32.9%)	1828 (28.7%)	<0.0001
No	5027 (67.3%)	96 (49.7%)	286 (52.4%)	225 (62.7%)	4420 (69.3%)	
Incarceration Past 6 Months						
Yes	1263 (16.9%)	65 (33.7%)*	184 (34.0%)*	73 (20.3%)	941 (14.8%)	<0.0001
No	5870 (78.5%)	115 (59.6%)	344 (63.0%)	262 (73.0%)	5149 (80.8%)	
Sexual Behavior						
Heterosexual ^b^	5495 (73.5%)	134 (69.4%)	382 (70.0%)	235 (65.5%)	4744 (74.4%)	0.1908
MSM	189 (2.5%)	12 (6.2%)*	11 (2.0%)	32 (8.9%)*	134 (2.1%)	<0.0001
WSW	203 (2.8%)	8 (4.1%)	30 (5.6%)*	13 (3.6%)	152 (2.4%)	<0.0001
Transactional Sex	726 (9.7%)	49 (25.4%)*	86 (15.8%)*	53 (14.8%)	538 (8.4%)	<0.0001
* Male*	*676 (9.0%)*	*44 (22.8%)*	*72 (13.2%)*	*49 (13.6%)*	*511 (8.0%)*	
* Female*	*50 (0.7%)*	*5 (2.6%)*	*14 (2.6%)*	*4 (1.1%)*	*27 (0.4%)*	
* Heterosexual*	*565 (7.6%)*	*38 (19.7%)*	*65 (11.9%)*	*40 (11.1%)*	*422 (6.6%)*	
* MSM*	*18 (0.2%)*	*1 (0.5%)*	*3 (0.5%)*	*4 (1.1%)*	*10 (0.2%)*	
Sex Solicitation	591 (7.9%)	57 (29.5%)*	125 (22.9%)*	47 (13.1%)*	362 (5.7%)	<0.0001
* Male*	187 (2.5%)	*28* (14.5%)	*30* (5.5%)	*18 (5.0*%)	*111* (1.7%)	
* Female*	404 (5.4%)	*29* (15.0%)	*95* (17.4%)	*29 (8.1*%)	*251* (3.9%)	
* Heterosexual*	438 (5.9%)	*43* (22.2%)	*92* (16.8%)	*28 (7.8*%)	*275* (4.3%)	
* MSM*	29 (0.4%)	*1* (0.5%)	*5* (0.9%)	*7 (1.9*%)	*16* (0.3%)	
Domestic Violence History	1047 (14.0%)	44 (22.8%)*	143 (26.1%)*	54 (15.0%)	806 (12.6%)	<0.0001
* Male*	303 (4.1%)	*19* (9.8%)	*42* (7.7%)	*16 (4.5*%)	*226* (3.5%)	
* Female*	744 (10.0%)	*25* (13.0%)	*101* (18.5%)	*38 (10.6*%)	*580* (9.1%)	
Sexual Assault History	698 (9.3%)	36 (18.7%)*	114 (20.9%)*	36 (10.0%)	512 (8.0%)	<0.0001
* Male*	130 (1.7%)	*11* (5.7%)	*21* (3.8%)	*11 (3.1*%)	*87* (1.4%)	
* Female*	568 (7.6%)	*25* (13.0%)	*93* (17.0%)	*25 (7.0*%)	*425* (6.7%)	
Previous STI						
Yes	1854 (24.8%)	73 (37.8%)	138 (25.2%)	115 (32.0%)*	1528 (24.0%)	<0.0001
Syphilis	396 (5.3%)	19 (9.8%)	42 (7.7%)*	34 (9.5%)	301 (4.7%)	<0.0001
No	5619 (75.1%)	120 (62.2%)	408 (74.7%)	244 (68.0%)	4847 (76.0%)	
Mental Health Diagnosis History						
Yes	1710 (22.9%)	105 (54.4%)*	244 (44.7%)*	107 (29.8%)	1254 (19.7%)	<0.0001
No	5763 (77.1%)	88 (45.6%)	302 (55.3%)	252 (70.2%)	5121 (80.3%)	

(N = 7473).

a P-value reported using the Kruskal-Wallis test; ^b^ P-value not significant using Dunn's multiple corrections test.

* p<0.05 comparing the non-infected group with each of the other groups using Dunn's multiple corrections test.

Legend: HIV  =  Human Immunodeficiency Virus; HCV  =  Hepatitis C Virus; MSM  =  Men-Who-Have-Sex-with-Men; WSW  =  Women-Who-Have-Sex-with-Women; STI  =  Sexually Transmitted Infection; PWID  =  Person Who Injects Drugs; “Speedball”  =  Injected Mixture of Cocaine and Heroin.

Bivariate analysis of HIV/HCV coinfection (Table 2) demonstrates an increased association of the coinfected group for a number of variables when compared to the HCV and HIV monoinfected groups. The magnitude of the correlation for several variables associated with the HIV/HCV coinfected group was higher than the monoinfected groups relative to being from the “baby boomer” generation (OR 8.78, p<0.00001), being unemployed (OR 9.77, p<0.0001), PWIDs (OR 73.86, p<0.0001), ever using a shooting gallery (OR 5.85, p<0.0001), or having been incarcerated in the previous 6 months (OR 3.09, p<0.0001). Not only does the HIV/HCV coinfected group have the highest OR of having used heroin, cocaine, and crack cocaine, but these individuals also have the highest OR of being opioid dependent, accessing opioid replacement therapy, and emergency department use in the past six months.

**Table 2:** Bivariate logistic regression comparisons of HIV/HCV coinfection, HCV monoinfection, and HIV monoinfection compared to patients without infection.

	HIV/HCV coinfected OR (CI)	p-value	HCV monoinfected OR (CI)	p-value	HIV monoinfected OR (CI)	p-value
Age (Years)	**1.07 (1.06–1.08)**	**<0.0001**	**1.04 (1.03–1.04)**	**<0.0001**	**1.03 (1.02–1.04)**	**<0.0001**
“Baby Boomer” Generation	**8.78 (6.29–12.25)**	**<0.0001**	**2.53 (2.12–3.02)**	**<0.0001**	**2.32 (1.87–2.88)**	**<0.0001**
Gender						
Male	**1.97 (1.44–2.70)**	**<0.0001**	**1.36 (1.13–1.62)**	**0.0010**	1.06 (0.86–1.32)	0.5650
Female	Referent		Referent		Referent	
Race/Ethnicity						
White	Referent		Referent		Referent	
Black	0.80 (0.57–1.14)	0.0700	**0.15 (0.11–0.19)**	**<0.0001**	**1.57 (1.17–2.11)**	**0.0020**
Hispanic	0.71 (0.49–1.03)	0.0700	**0.43 (0.36–0.53)**	**<0.0001**	1.12 (0.82–1.54)	0.4840
Other	1		**0.17 (0.04–0.70)**	**0.0150**	1.52 (0.54–4.32)	0.4290
U.S. Born						
Yes	**2.02 (1.38–2.94)**	**<0.0001**	**3.95 (2.96–5.29)**	**<0.0001**	0.88 (0.70–1.10)	0.2840
No	Referent		Referent		Referent	
Did Not Graduate High School						
Yes	**1.71 (1.27–2.31)**	**<0.0001**	1.18 (0.98–1.43)	0.0870	**1.26 (1.01–1.59)**	**0.0450**
No	Referent		Referent		Referent	
Unstable Housing						
Yes	**3.20 (2.39–4.28)**	**<0.0001**	**3.8 (3.17–4.55)**	**<0.0001**	**1.32 (1.05–1.65)**	**0.0170**
No	Referent		Referent		Referent	
Not in a Relationship						
Yes	**1.76 (1.14–2.72)**	**<0.0100**	**1.93 (1.48–2.53)**	**<0.0001**	0.79 (0.62–1.02)	0.0660
No	Referent		Referent		Referent	
Unemployed						
Yes	**9.77 (5.15–18.52)**	**<0.0001**	**4.71 (3.56–6.22)**	**<0.0001**	**1.88 (1.46–2.43)**	**<0.0001**
No	Referent		Referent		Referent	
Health Insurance						
Yes	**3.19 (2.33–4.36)**	**<0.0001**	**4.15 (3.40–5.06)**	**<0.0001**	**1.58 (1.28–1.96)**	**<0.0001**
No	Referent		Referent		Referent	
Government Assistance						
Yes	**4.53 (3.39–6.07)**	**<0.0001**	**3.71 (3.11–4.44)**	**<0.0001**	**1.34 (1.06–1.69)**	**0.0160**
No	Referent		Referent		Referent	
Injection Drug Use (PWID)						
Yes	**73.86 (47.74–114.25)**	**<0.0001**	**44.05 (35.0–55.4)**	**<0.0001**	**2.54 (1.93–3.35)**	**<0.0001**
No	Referent		Referent		Referent	
Injection Drug Use Last 30 Days:						
Yes	**9.78 (6.77–14.14)**	**<0.0001**	**12.8 (10.1–16.3)**	**<0.0001**	**2.18 (1.32–3.60)**	**<0.0020**
No	Referent		Referent		Referent	
Non-injection Drug Use, excluding Marijuana						
Yes	0.79 (0.32–1.94)	0.6120	**0.50 (0.25–0.98)**	**0.0430**	1.57 (0.96–2.58)	0.0720
No	Referent		Referent		Referent	
Injected with Used Needles						
Yes	**3.94 (2.44–6.37)**	**<0.0001**	**3.50 (2.54–4.83)**	**<0.0001**	1.64 (0.75–3.58)	0.2190
No	Referent		Referent		Referent	
Shooting Gallery Use						
Yes	**5.85 (3.29–10.38)**	**<0.0001**	**3.65 (2.42–5.50)**	**<0.0001**	1.90 (0.62–5.83)	0.2600
No	Referent		Referent		Referent	
Heroin Use						
Yes	**39.08 (25.2–60.72)**	**<0.0001**	**33.20 (25.79–42.75)**	**<0.0001**	**2.52 (2.00–3.18)**	**<0.0001**
No	Referent		Referent		Referent	
Cocaine Use						
Yes	**22.32 (14.00–35.56)**	**<0.0001**	**13.25 (10.49–16.73)**	**<0.0001**	**2.17 (1.75–2.69)**	**<0.0001**
No	Referent		Referent		Referent	
Crack Cocaine Use						
Yes	**16.03 (11.02–23.32)**	**<0.0001**	**12.17 (9.85–15.02)**	**<0.0001**	**2.92 (2.35–3.62)**	**<0.0001**
No	Referent		Referent		Referent	
Marijuana Use						
Yes	**5.80 (3.67–9.14)**	**<0.0001**	**5.24 (4.02–6.82)**	**<0.0001**	**1.50 (1.20–1.88)**	**<0.0001**
No	Referent		Referent		Referent	
Combined Cocaine and Heroin (“Speedball”) Use						
Yes	**32.04 (23.22–44.20)**	**<0.0001**	**15.87 (13.06–19.28)**	**<0.0001**	**3.18 (2.39–4.22)**	**<0.0001**
No	Referent		Referent		Referent	
Crystal Methamphetamine Use						
Yes	**7.65 (4.99–11.74)**	**<0.0001**	**8.43 (6.36–11.16)**	**<0.0001**	1.50 (0.82–2.72)	0.1870
No	Referent		Referent		Referent	
MDMA (“Ecstasy”) Use						
Yes	0.998 (0.62–1.61)	0.9920	**2.43 (1.95–3.04)**	**<0.0001**	0.86 (0.59–1.26)	0.4450
No	Referent		Referent		Referent	
Hazardous Drinking						
Yes	1.05 (0.729–1.51)	0.7980	0.79 (0.62–1.01)	0.0600	1.15 (0.88–1.49)	0.3050
No	Referent		Referent		Referent	
Opioid Dependence Use						
Yes	**12.01 (8.86–16.30)**	**<0.0001**	**7.07 (5.72–8.74)**	**<0.0001**	**2.81 (2.06–3.84)**	**<0.0001**
No	Referent		Referent		Referent	
Opioid Substitution Therapy Use						
Yes	**16.45 (12.12–23.33)**	**<0.0010**	**10.82 (8.78–13.34)**	**<0.0001**	**3.17 (2.31–4.37)**	**<0.0001**
No	Referent		Referent		Referent	
Emergency Room Past 6 Months						
Yes	**2.17 (1.61–2.91)**	**<0.0001**	**2.12 (1.78–2.54)**	**<0.0001**	**1.27 (1.01–1.59)**	**0.0420**
No	Referent		Referent		Referent	
Incarceration Past 6 Months						
Yes	**3.09 (2.26–4.23)**	**<0.0001**	**2.93 (2.42–3.55)**	**<0.0001**	**1.52 (1.17–1.99)**	**0.0020**
No	Referent		Referent		Referent	
Sexual Behavior						
Heterosexual	Referent		Referent		Referent	
MSM	**3.32 (1.79–6.14)**	**<0.0001**	1.04 (0.56–1.95)	0.893	**4.94 (3.29–7.44)**	**<0.0001**
WSW	**1.96 (0.86–3.66)**	**0.0710**	2.53 (1.68–3.79)	**<0.0001**	1.78 (1.00–3.19)	0.0510
Transactional Sex						
Yes	**7.20 (5.18–10.02)**	**<0.0001**	**4.93 (3.93–6.19)**	**<0.0001**	**2.59 (1.87–3.58)**	**<0.0001**
No	Referent		Referent		Referent	
Sex Solicitation						
Yes	**3.80 (2.71–5.33)**	**<0.0001**	**2.02 (1.58–2.59)**	**<0.0001**	**1.92 (1.41–2.60)**	**<0.0001**
No	Referent		Referent		Referent	
Domestic Violence						
Yes	**1.93 (1.35–2.75)**	**<0.0001**	**2.33 (1.89–2.88)**	**<0.0001**	**1.69 (1.23–2.32)**	**0.0010**
No	Referent		Referent		Referent	
Sexual Assault						
Yes	**2.49 (1.70–3.65)**	**<0.0001**	**2.87 (2.28–3.61)**	**<0.0001**	**1.72 (1.19–2.49)**	**0.0040**
No	Referent		Referent		Referent	
Sexually Transmitted Infection						
Yes	**1.93 (1.43–2.60)**	**<0.0001**	**1.07 (0.88–1.31)**	**0.4920**	**1.50 (1.19–1.88)**	**0.0010**
No	Referent		Referent		Referent	
Syphilis						
Yes	**2.20 (1.35–3.59)**	**<0.0001**	**1.68 (1.20–2.35)**	**0.0002**	**2.11 (1.46–3.06)**	**<0.0001**
No	Referent		Referent		Referent	
Mental Health Diagnosis						
Yes	**4.87 (3.65–6.51)**	**<0.0001**	**3.30 (2.76–3.95)**	**<0.0001**	**1.73 (1.37–2.19)**	**<0.0001**
No	Referent		Referent		Referent	

HIV  =  Human Immunodeficiency Virus; HCV  =  Hepatitis C Virus; MSM  =  Men-Who-Have-Sex-with-Men; WSW  =  Women-Who-Have-Sex-with-Women; STI  =  Sexually Transmitted Infection; PWID  =  Person Who Injects Drugs; “Speedball”  =  Injected Mixture of Cocaine and Heroin.

HCV monoinfection had higher association for women having sex with women (OR 2.53, p<0.0001) and interpersonal violence (IPV) of both genders: domestic violence (OR 2.33, p<0.0001) and sexual assault (OR 2.87, p<0.0001). The HCV monoinfected group was also more likely to have used MDMA “ecstasy” (OR 2.43, p<0.0001) as well having higher levels of drug injection in the previous thirty days (OR 12.8, p<0.0001). MSM were more likely to be in the HIV monoinfection category (OR 4.94, p<0.0001). Additional comparisons of the non-infected group with each of the three infection groups are denoted using an asterisk (*) when p<0.05. Notably, for some variables, not each infection group was significantly different than the non-infected group.

**Figure 1:**
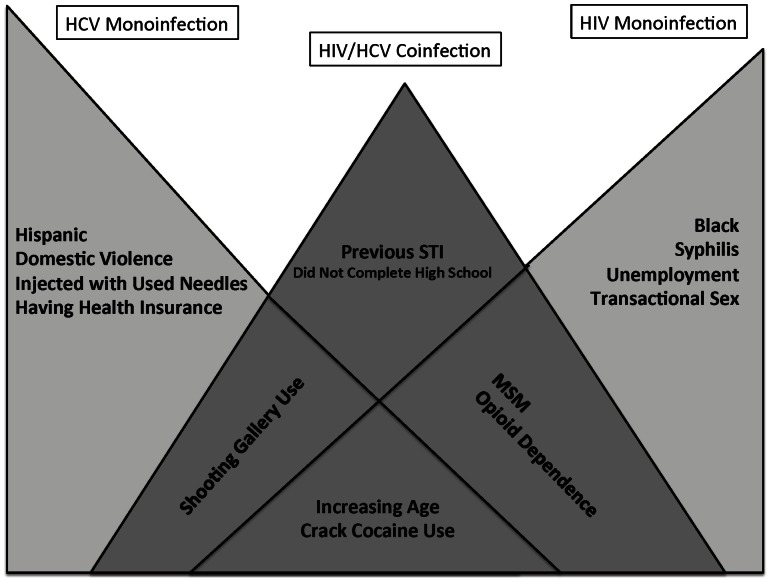
Graphical Representation of Overlapping Relationships Between HIV/HCV Coinfection, HCV Monoinfection and HIV Monoinfection, 2003-2011. (N = 7473) MSM  =  Men-Who-Have-Sex-with-Men; STI  =  Sexually Transmitted Infection.

The comprehensive multivariate model findings with the best-fit models (Table 3), and the graphic depiction of correlates of each of the three infection statuses are depicted (Figure 1). Please note the figure is not drawn to scale to reflect the magnitude of the associations but only to demonstrate where significant differences exist. All three infection groups were independently correlated with increasing age and crack cocaine use. HIV/HCV coinfection was independently correlated with ever visited a shooting gallery, meeting criteria for opioid dependence, previous STI, being MSM, and not completing high school. The HIV/HCV coinfection model intersects with the HCV monoinfected model with ever visiting a shooting gallery and intersects with the HIV-monoinfection model with being MSM or opioid dependent. In addition to those characteristics shared by the HIV/HCV coinfection group, HCV monoinfection is characterized by being Hispanic, PWIDs, history of domestic violence, and having health insurance. HIV monoinfection is characterized by being Black, transactional sex, having had syphilis, and a history of unemployment.

**Table 3:** Multivariate analysis with best fit models for HIV/HCV coinfection using both forwards and backwards regression modeling with AIC and Pearson's correlation for bivariate correlations with p<.05.

HIV/HCV coinfected			HCV monoinfected			HIV monoinfected		
Covariate	AOR (CI)	p-value	Covariate	AOR (CI)	p-value	Covariate	AOR (CI)	p-value
**Increasing Age**	1.15 (1.10–1.20)	<0.0001	**Increasing Age**	1.07 (1.05–1.10)	<0.0001	**Increasing Age**	1.03 (1.02–1.04)	<0.0001
**Crack Cocaine Use**	4.72 (1.23–18.09)	0.0240	**Crack Cocaine Use**	1.82 (1.01–3.29)	0.0460	**Crack Cocaine Use**	1.69 (1.21–2.37)	0.0020
**Shooting Gallery Use**	3.06 (1.36–6.87)	0.0070	**Shooting Gallery Use**	1.89 (1.13–3.16)	0.0150	**Transactional Sex**	1.65 (1.07–2.55)	0.0250
**MSM**	38.53 (2.58–575.80)	0.0080	**Injected with Used Needles**	2.15 (1.32–3.51)	0.0020	**MSM**	6.59 (4.05–10.74)	<0.0001
**Opioid** **Dependence**	3.90 (1.73–8.82)	0.0010	**Domestic Violence**	1.99 (1.21–3.28)	0.0070	**Opioid Dependence**	6.59 (4.05–10.74)	<0.0001
**Previous STI**	3.31 (1.40–7.81)	0.0060	**Having Health Insurance**	2.16 (1.32–3.54)	0.0020	**Syphilis**	2.66 (1.73–4.09)	<0.0001
**Did Not Complete** **High School**	2.51 (5.93–1.06)	0.0370	**Hispanic Ethnicity**	2.63 (1.58–4.38)	<0.0001	**Black**	1.73 (1.28–2.34)	0.0010
		**AIC 177.83**			**AIC 482.09**			**AIC 1502.61**
**Pearson**'**s Chi^2^ = 160.37** **prob > chi2 = 0.9738**		**BIC 206.00**	**Pearson**'**s Chi^2^ = 359.82** **prob > chi2 = 0.2092**		**BIC 544.52**	**Pearson**'**s Chi^2^ = 851.61** **prob > chi2 = 0.5551**		**BIC 1560.68**

Hepatitis C and HIV monoinfected groups are included for comparison.

HIV  =  Human Immunodeficiency Virus; HCV  =  Hepatitis C Virus; MSM  =  Men-Who-Have-Sex-with-Men; WSW  =  Women-Who-Have-Sex-with-Women; STI  =  Sexually Transmitted Infection; AIC  =  Akaike Information Criteria; BIC =  Bayesian Information Criterion.

**Figure 2:**
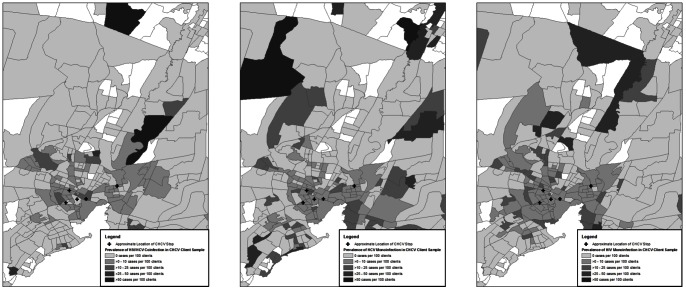
Geospatial density of HIV/HCV Coinfection, HCV Monoinfection, and HIV Monoinfection, respectively, in CHCV population by Census Block Group, 2003-2011. (N = 7,720).

Prevalence mapping for HIV/HCV coinfection, HCV monoinfection, and HIV monoinfection showed the spatial distribution of the three respective infection groups within the study population (Figure 2). The selected area represents 430 census block groups in the greater New Haven, CT area and includes a total 7,068 CHCV clients with full demographic information (94.8%). This area includes 185 of the HIV/HCV coinfected clients (95.9%), 483 of the HCV monoinfected clients (88.5%), and 330 of the HIV monoinfected clients (91.9%). Per census block group, there is a mean of 0.45 HIV/HCV coinfected, 1.19 HCV monoinfected, and 0.81 HIV monoinfected clients. As shown in Figure 2, regions of highest prevalence among the three infections have distinct geographical prevalence patterns.

## Discussion

To our knowledge, this study represents the first attempt to disaggregate the correlates of HIV and HCV monoinfection and HIV/HCV coinfection in an urban Northeast setting. The study sample included individuals accessing care in a mobile health setting that is profoundly impacted by both infections. As such, it provides valuable insight into community-based assessments not included in household surveys, since most of the participants accessing healthcare did not live in stable housing settings and would not be included using standard survey methods. Moreover, this assessment includes individuals who are seeking healthcare. The added granularity found in this analysis suggests that the correlates of HIV/HCV coinfection in such an urban setting is not just a magnification of known risk factors such as drug injection, sex work, and incarceration but provides added contribution of social, medical and risk behavior comorbidity among a diverse population of foreign-born, MSM, women having sex with women, people of color, the unemployed, and victims of IPV that may contribute independently to HIV or HCV infection, respectively, or both.

In this setting, HCV infection in HIV infected individuals was found to be 32.1%, which is similar to previously reported studies [3] and reflects a high risk population in this urban, Northeastern community. Though there may be a collection bias in this study population, these numbers reflect similar high-risk communities nationally and thus may have implications for other high-risk groups elsewhere in settings where there is a high prevalence of substance use disorders. Further, as this study shows that HIV/HCV coinfection is correlated with increasing age, this has implications for the need to enhance HCV testing in community settings with subsequent referral to treatment before development of non-reversible, end-stage liver disease or hepatocellular carcinoma.

Our data reinforce growing evidence of the connection between HCV infection and IPV, which in one prospective study found a five-fold increase in the likelihood of having HCV even after controlling for past injection drug use [11]. While previous studies have documented IPV as an independent risk for HIV acquisition according to the SAVA syndemic (**S**ubstance **A**buse, **V**iolence, and HIV/**A**IDS) [35], our data show that IPV imparts more than two-fold increased association for HCV infection. Though our study does not affirm causality, it does have important implications for screening and treatment since IPV is associated with increased risk behaviors and poor retention in care [35].

This study also contributes significantly to a better community understanding of HIV, HCV, and HIV/HCV coinfection geography. Our spatial analysis confirms that these infections vary in their geographical distribution. While HIV/HCV coinfection seems to be distributed equally among ethnic groups, HCV monoinfection has a higher correlation in the Hispanic community, and HIV monoinfection has higher correlation in both Black and MSM communities in this population. HIV/HCV does not merely represent the potentiation of HCV and HIV risk factors but rather an intersection of other factors (i.e., shooting gallery use) and high-risk sexual behaviors that differ from the HCV and HIV monoinfected groups. Thus, communities may consider targeted screening in settings where such social and medical comorbidities exist to increase access to treatment for this population, who if left untreated, will eventually develop end-stage liver disease, hepatocellular carcinoma and potentially require costly transplantation.

As the association between MSM and syphilis is well-established, this study serves to corroborate this correlation in our study population [36]. Further, the markedly high association portending nearly a 40-fold association between being MSM and having HIV/HCV coinfection is concerning and may reflect recent reports of acute HCV transmission within the HIV-infected MSM community both nationally and internationally [37], [38].

The high correlation of crack cocaine use among all three infection groups is an understudied comorbidity likely correlated with high-risk sexual transmission and thus deserves greater attention for behavioral, educational, and programmatic public health interventions**.** Moreover, crack cocaine use has important implications for HIV and HCV treatment, including problematic access to and retention in care and adherence to antiretroviral therapy [39].

The 2012 U.S. Congressional Budget Office report estimated that approximately 55 million (1 of 5) Americans under age 65 did not have any type of health insurance [40], which equates to poor access to screening and treatment for HIV and/or HCV infection. Similarly, the U.S. Department of Health and Human Services estimates that 24% of PLWHA are currently uninsured despite the Ryan White AIDS Care Act and AIDS Drug Assistance Programs (ADAP) [41]. While ADAP waiting lists have been reduced to 694 individuals in seven states as of August 2012 [42], the future of such funding is not known. Though the Patient Protection and Affordable Care Act will provide added protection to PLWHA by eliminating discrimination from pre-existing conditions, the extent to which it expands Medicaid coverage is wholly dependent on the unpredictable mandates of state legislatures. The inconsistent status of state-operated ADAP program coverage has been emblematic of non-uniform treatment of PLWHA. Thus, HIV/HCV coinfected individuals, especially those who have variable incomes or have interrupted insurance eligibility due to repeated incarceration, will continue to experience problematic healthcare access and remain at the margins of treatment. While the ACA will not cover medical services for undocumented immigrants [43], data from this study do not suggest that this group is at increased need for screening and treatment, though large scale immigration to New Haven from Latin America is relatively recent.

Not surprising is the common relationship between injection drug use, especially shooting galleries where injecting equipment is often shared, and HIV, HCV, and HIV/HCV infections. The most recent guidance about the future of HIV prevention by the U.S. CDC omitted needle and syringe exchange programs (NSEPs) from its list of prevention packages [44]. While this may reflect the government's unwillingness to support and fund such programs that have markedly reduced HIV transmission in communities, its omission is remarkably salient since such programs have benefits beyond HIV transmission [15], [19], [45].

Though this study highlights a number of new and important findings, it is, however, limited by a number of factors. First, most but not all patients were systematically screened for HIV and HCV, reducing the reliability of comparison to the coinfected group that was clearly screened for both infections. Though HIV testing was routine, HCV testing was risk-based. Also, the cross-sectional nature of this clinical sample can only demonstrate association and does not prove causation. As a result, such findings should be incorporated into future prospective cohort studies. Although the clinical intake questionnaire was clinically comprehensive, it did not fully assess all potential HCV transmission risk factors such as detailed tattooing practices (e.g. within prison), unprotected sex with someone infected with HCV, or ever having been incarcerated, though recent incarceration was assessed.

Notwithstanding these limitations, this large clinical sample included a considerable number of high risk individuals who are medically and socially marginalized and thus allowed us to use a real-world patient population to assess screening and treatment priorities for urban communities with markedly increased health disparities, including HCV, HIV, and HIV/HCV coinfection.

### Conclusions

This study uses a community-wide approach using real-world clinical data and geospatial mapping to address HCV and HIV monoinfection and HIV/HCV coinfection in a heterogeneously, urban, at-risk Northeastern population. Of note, the correlates and geospatial mapping suggest a number of different characteristics associated with mono- and dual-infection. These differences have important implications for screening, linkage to care, and provision of treatment for these diverse populations that share traditionally common risk factors. Communities similar to this one that struggles with considerable health disparities may use similar approaches in planning community-based activities for at risk, vulnerable populations.
